# Childhood trauma, brain structure and emotion recognition in patients with schizophrenia and healthy participants

**DOI:** 10.1093/scan/nsaa160

**Published:** 2020-11-27

**Authors:** Karolina I Rokita, Laurena Holleran, Maria R Dauvermann, David Mothersill, Jessica Holland, Laura Costello, Ruán Kane, Declan McKernan, Derek W Morris, John P Kelly, Aiden Corvin, Brian Hallahan, Colm McDonald, Gary Donohoe

**Affiliations:** School of Psychology, National University of Ireland Galway, Galway, Ireland; Centre for Neuroimaging, Cognition & Genomics, National University of Ireland Galway, Galway, Ireland; School of Psychology, National University of Ireland Galway, Galway, Ireland; Centre for Neuroimaging, Cognition & Genomics, National University of Ireland Galway, Galway, Ireland; School of Psychology, National University of Ireland Galway, Galway, Ireland; Centre for Neuroimaging, Cognition & Genomics, National University of Ireland Galway, Galway, Ireland; Department of Brain and Cognitive Sciences, McGovern Institute for Brain Research, MIT, Cambridge, MA 02135, USA; School of Psychology, National University of Ireland Galway, Galway, Ireland; Centre for Neuroimaging, Cognition & Genomics, National University of Ireland Galway, Galway, Ireland; School of Business, National College of Ireland, Dublin, Ireland; School of Psychology, National University of Ireland Galway, Galway, Ireland; Centre for Neuroimaging, Cognition & Genomics, National University of Ireland Galway, Galway, Ireland; Centre for Neuroimaging, Cognition & Genomics, National University of Ireland Galway, Galway, Ireland; School of Psychology, National University of Ireland Galway, Galway, Ireland; Pharmacology & Therapeutics, School of Medicine, National University of Ireland Galway, Galway, Ireland; Centre for Neuroimaging, Cognition & Genomics, National University of Ireland Galway, Galway, Ireland; Pharmacology & Therapeutics, School of Medicine, National University of Ireland Galway, Galway, Ireland; Department of Psychiatry, Trinity Centre for Health Sciences, St. James’s Hospital, Dublin, Ireland; Centre for Neuroimaging, Cognition & Genomics, National University of Ireland Galway, Galway, Ireland; Department of Psychiatry, Clinical Science Institute, National University of Ireland Galway, Galway, Ireland; Centre for Neuroimaging, Cognition & Genomics, National University of Ireland Galway, Galway, Ireland; Department of Psychiatry, Clinical Science Institute, National University of Ireland Galway, Galway, Ireland; School of Psychology, National University of Ireland Galway, Galway, Ireland; Centre for Neuroimaging, Cognition & Genomics, National University of Ireland Galway, Galway, Ireland

**Keywords:** childhood trauma, social cognition, emotion recognition, brain structure, schizophrenia

## Abstract

Childhood trauma, and in particular physical neglect, has been repeatedly associated with lower performance on measures of social cognition (e.g. emotion recognition tasks) in both psychiatric and non-clinical populations. The neural mechanisms underpinning this association have remained unclear. Here, we investigated whether volumetric changes in three stress-sensitive regions—the amygdala, hippocampus and anterior cingulate cortex (ACC)—mediate the association between childhood trauma and emotion recognition in a healthy participant sample (*N* = 112) and a clinical sample of patients with schizophrenia (*N* = 46). Direct effects of childhood trauma, specifically physical neglect, on Emotion Recognition Task were observed in the whole sample. In healthy participants, reduced total and left ACC volumes were observed to fully mediate the association between both physical neglect and total childhood trauma score, and emotion recognition. No mediating effects of the hippocampus and amygdala volumes were observed for either group. These results suggest that reduced ACC volume may represent part of the mechanism by which early life adversity results in poorer social cognitive function. Confirmation of the causal basis of this association would highlight the importance of resilience-building interventions to mitigate the detrimental effects of childhood trauma on brain structure and function.

## Introduction

The ability to recognise emotional facial expressions is a highly developed and integral feature of social cognition, and an important predictor of socio-occupational functioning, contributing to quality of life (Fett *et al.*, 2011; Fiszdon *et al.*, 2013). Impairments in emotion recognition have been particularly well-documented in psychiatric disorders, such as schizophrenia (SZ), which is characterised by wide-ranging social cognitive deficits (Mier and Kirsch, 2017). Individual differences in emotion recognition have also been found in healthy samples with lower age and higher education level leading to a better ability to recognise facial expressions (Isaacowitz *et al.*, 2007; Tamamiya and Hiraki, 2013).

While adverse childhood experiences (i.e. emotional abuse, physical abuse, sexual abuse, emotional neglect and physical neglect) have been consistently associated with various negative outcomes across the life course, including mental health problems (Bendall *et al.*, 2008; Mandavia *et al.*, 2016; Morgan and Gayer-Anderson, 2016; Hébert *et al.*, 2018), there is also growing evidence that traumatic experiences in childhood are related to deficits in the ability to recognise emotions in both clinical (e.g. SZ) and non-clinical populations (Rokita *et al.*, 2018). Although childhood trauma is relatively common in the general population [30%; (Kessler *et al.*, 2010)], its prevalence is particularly high in individuals with SZ [85% (Larsson *et al.*, 2013)], with physical and emotional neglect being the most prevalent forms of child maltreatment (Stoltenborgh *et al.*, 2013). In previous studies, we explored the possible cognitive mechanisms by which traumatic experiences in childhood may lead to social cognitive deficits in later life, in both clinical and non-clinical populations, suggesting the mediating role of attachment-related processes (Rokita *et al.*, 2018, 2020). However, the neural substrates underlying this relationship have yet to be clarified as the specific association between childhood trauma, brain structure and social cognitive function has not yet been investigated.

Across both clinical and non-clinical samples, neuroimaging studies have found evidence that childhood trauma is associated with variability on multiple measures of brain structure and function (Heany *et al.*, 2017; Cancel *et al.*, 2019). On measures of brain structure, experience of childhood trauma has been linked to reductions in total grey matter volume (Cancel *et al.*, 2015; Frissen *et al.*, 2018; Lim *et al.*, 2018) and volume reduction primarily in the hippocampus, amygdala, anterior cingulate cortex (ACC) and dorsolateral and ventromedial prefrontal cortices (PFCs) (Teicher *et al.*, 2016; Cancel *et al.*, 2019; Popovic *et al.*, 2019) (see Supplementary Table S1 for a review of studies). Moreover, childhood trauma has been associated with changes in brain activity during emotional face tasks, mostly in the hippocampus, amygdala and ACC (Benedetti *et al.*, 2011; Anticevic *et al.*, 2012; van Harmelen *et al.*, 2013; Teicher and Samson, 2016; Quide *et al.*, 2017) as well as decreased functional connectivity between limbic (i.e. amygdala and hippocampus) and cortical (PFC and ACC) regions (Herringa *et al.*, 2013; Cancel *et al.*, 2017; Kraynak *et al.*, 2019). However, brain activation during emotional face processing in individuals with high levels of childhood trauma appears to be dependent on emotional valence of faces. For instance, Aas *et al.* (2017) observed negativity bias in the processing of emotional faces and stronger differentiation in brain responses in a sample of patients with SZ spectrum and bipolar spectrum diagnosis, with clusters comprising the right angular gyrus, supramarginal gyrus, middle temporal gyrus and lateral occipital cortex.

Given the evidence that structural brain alterations in maltreated individuals are primarily observed in brain areas relevant for emotion processing, such as the amygdala, hippocampus and ACC, this is highly suggestive of the possible mediating effects of these structural alterations in the relationship between childhood trauma and emotion recognition.

While emotion processing has been linked to multiple brain structures and areas, including the fusiform face area, amygdala, insula, hippocampus, medial prefrontal cortex and ACC (Fujiwara *et al.*, 2007; Adolphs, 2009; Anticevic *et al.*, 2012; Maat *et al.*, 2016; Szymkowicz *et al.*, 2016; Krautheim *et al.*, 2018) in both structural and functional imaging studies, this study will focus on three regions of interest (ROIs)—namely, the amygdala, hippocampus and ACC—as these structures show particular sensitivity to the effects of childhood trauma.

Despite substantial evidence for the impact of childhood trauma on brain structures involved in social cognition, particularly emotion recognition, the mediating effects of these regional brain alterations have never been investigated in psychiatric or healthy samples, to the best of our knowledge. The main objective of this study was to investigate the relationship between childhood trauma, brain structure and emotion recognition in both healthy participants and a clinical sample of patients with SZ to confirm the possible differential effects of adversity on regional brain volume and the ability to recognise emotions. Based on our recent systematic review and findings (Rokita *et al.*, 2018, 2020), we tested the hypothesis that the association between higher levels of childhood trauma and poorer emotion recognition would be mediated via reduced volumes in one or more of three stress-sensitive brain regions—the hippocampus, amygdala and ACC.

## Methods

### Participants

The current study was part of the ‘Immune Response & Social Cognition in Schizophrenia’ (‘iRELATE’) research project funded by the European Research Council examining the impact of the environment, genes and immune system on brain structure and function in SZ. Data were derived from 112 healthy participants (81 males and 31 females) and 46 patients (34 males and 12 females) either with a diagnosis of SZ (*N* = 35) or schizoaffective disorder (SZA) (*N* = 11). Patients with SZ and SZA were included in the same group as they exhibit a similar pattern of cognitive impairments and structural brain abnormalities (Amann *et al.*, 2016; Hartman *et al.*, 2019). Healthy participants were recruited through adverts placed in the Galway and Dublin areas, and patients were recruited from the local outpatient clinics and mental health services (e.g. day centres and day hospitals).

Inclusion criteria for patients were the following: (i) a diagnosis of SZ or SZA as confirmed by the Structured Clinical Interview for DSM-IV (Diagnostic and Statistical Manual of Mental Disorders, 4th Edition) (SCID) (American Psychiatric Association, 2000); (ii) being clinically stable at the time of assessment (i.e. medicated and being an outpatient) (iii) and no report of co-morbid psychiatric disorders. Healthy participants were only included in the study if they met the following criteria: (i) no report of mental or general health problems; (ii) no use of antipsychotic medication and (iii) having no first-degree relative with SZ or SZA. All participants were Caucasian and had to be aged between 18 and 65 years. Exclusion criteria for both groups were as follows: (i) a history of acquired brain injury causing loss of consciousness of >1 minute; (ii) substance abuse in the preceding 6 months; (iii) intellectual disability (i.e. IQ < 70); (iv) magnetic resonance imaging (MRI) contra-indicators (e.g. metal implants and claustrophobia) and (v) and a neurological disorder (e.g. epilepsy). Demographic and clinical characteristics of patients and healthy participants are presented in Table 1.

**Table 1.** Sociodemographic and clinical characteristics of patients with SZ and healthy participants

	Patients (*N* = 46)	Healthy participants (*N* = 112)	Difference
Variable	Mean (s.d.)	Mean (s.d.)	*T/χ* ^2^
Age	43.74 (10.94)	40.13 (10.81)	−1.898
Gender (male: female)^a^	34:12	81:31	0.042
Years of education	15.04 (2.37)	17.28 (3.30)	4.157***
IQ estimate	95.30 (15.96)	111.40 (16.16)	5.709***
Duration of illness (in years)	17.86 (12.24)	N/A	N/A
Diagnosis (SZ:SZA)	35:11	N/A	N/A
Positive symptoms (PANSS)	1.61 (2.26)	N/A	N/A
Negative symptoms (PANSS)	2.64 (3.66)	N/A	N/A
General symptoms (PANSS)	4.43 (4.17)	N/A	N/A
Total score (PANSS)	8.45 (8.33)	N/A	N/A
Antipsychotic medication^b^^,^^c^ (mg)	337.07 (229.31)^d^	N/A	N/A
Childhood Trauma Questionnaire			
Emotional abuse	4.35 (5.00)	3.33 (4.10)	−1.327
Physical abuse^a^	1.85 (3.76)	1.54 (2.78)	0.538
Sexual abuse^a^	2.04 (4.85)	0.42 (1.45)	3.307
Emotional neglect	4.83 (4.77)	4.26 (4.11)	−0.584
Physical neglect	2.52 (3.16)	1.48 (2.37)	−2.231*
CTQ total score	15.59 (15.61)	11.04 (10.52)	−1.985*
ERT^e^			
ERT total score	25.52 (5.82)	30.36 (3.94)	6.056***

N/A: not applicable; PANSS, Positive and Negative Syndrome Scale; IQ, Wechsler Adult Intelligence Scale estimated Intelligence Quotient; ERT, Emotion Recognition Task.

*P < 0.05; *P* < 0.01; ***P < 0.001.

^a^ A chi-square test was conducted.

^b^ Chlorpromazine equivalent.

^c^ Antipsychotic medication included the following: aripiprazole, clozapine, olanzapine, paliperidone, quetiapine, risperidone and zuclopenthixol.

^d^ No significant differences were reported between patients with SZ and SZA (*P* = 0.542).

^e^ The ERT data were normally distributed for both groups, thus no ceiling effects were observed.

### Procedure

Following initial screenings conducted to identify eligible individuals to take part, participants were administered a comprehensive battery of measures over three study visits. The National University of Ireland Galway Research Ethics Committee, the Clinical Research Ethics Committee at University Hospital Galway and the Research Ethics Committee at Tallaght Hospital in Dublin reviewed and approved all study procedures. All participants provided written informed consent prior to the study visits.

### Measures

#### Clinical measures.

The severity of symptomatology in patients was measured using the Positive and Negative Syndrome Scale [PANSS; (Kay *et al.*, 1987)]. This 30-item semi-structured interview consists of three subscales: positive symptoms scale (7 items), negative symptoms scale (7 items) and general psychopathology scale (16 items). To reflect the ‘absence’ scores, we used the re-scaled Likert scale ranging from 0 (absent) to 6 (extreme). Ultimately, the total scores ranged from 0 to 42 for the positive and negative scales, from 0 to 96 for the general scale and from 0 to 138 for the total score.

#### Childhood trauma experiences.

The Childhood Trauma Questionnaire [CTQ; (Bernstein *et al.*, 2003)] was used assess traumatic experiences during childhood up to the age of 16. The CTQ is a 28-item self-report questionnaire investigating five different types of childhood trauma, including emotional abuse, physical abuse, sexual abuse, emotional neglect and physical neglect. Each scale consists of five items and a 5-point Likert scale is used for the responses, which range from 1 (never true) to 5 (very often true). However, in order to reflect the absence of trauma, we used the re-scaled Likert scale ranging from 0 (never true) to 4 (very often true). Ultimately, each subscale ranged from 0 to 20 and the total score ranged from 0 to 100, with higher scores indicating more traumatic experiences.

#### Emotion recognition.

Emotion recognition was assessed with the Emotion Recognition Task (ERT)—short version, which is a computerised task, implemented in the Cambridge Neuropsychological Test Automated Battery (Robbins *et al.*, 1994) evaluating the ability to recognise six basic emotions: happiness, sadness, disgust, anger, surprise and fear. The task includes five practice trials and 48 assessed trials of male and female facial expressions of eight different intensities. Participants are instructed to correctly identify emotions presented on the screen by choosing one out of six names of emotional expressions. The overall number of correct responses (for all emotions) is the main outcome of this task that we assess in the current study.

#### Neuropsychological assessment.

Three subtests from the Wec-hsler Adult Intelligence Scale—Third Edition [WAIS-III-R; (Wechsler, 1997)] including Vocabulary, Digit Symbol and Block Design were administered to estimate Full Scale Intelligence Quotient. The shortened versions of the WAIS-III are frequently used to reduce administration time and were previously found to highly correlate (*r* > 0.9) with the full version consisting of 11 subtests (Axelrod, 2002; Wymer *et al.*, 2003).

#### MRI acquisition.

Imaging for all participants was performed on a 3T Philips Achieva MR scanner (Philips Medical Systems, Best, the Netherlands), located in the Centre for Advanced Medical Imaging at St. James’s Hospital, Dublin, Ireland. Three-dimensional T1-weighted structural scans (FFE (Fast Field Echo) pulse sequence, TR (Repetition Time)/TE (Echo Time) = 8.5/3.9 ms, FOV (Field-of-view) = 256 × 256 × 160 mm^3^, a spatial resolution of 1 mm^3^, TI (Inversion Time) = 1060 ms, flip angle = 8°, SENSE (Sensitivity Encoding) factor = 1.5 and acquisition time = 7 min 30 s) of the whole brain were acquired for each participant. Foam padding was used to stabilise the head, and headphones were provided to minimise the scanner noise.

#### MRI analysis.

The FreeSurfer image analysis software suite [Version 6.0.0; https://surfer.nmr.mgh.harvard.edu/ (Fischl *et al.*, 2002)] was used for processing of the T1-weighted images, including cortical surface reconstruction, cortical volume parcellation and subcortical volume segmentation. The Desikan-Killiany atlas was used to label 34 cortical structures per hemisphere (Desikan *et al.*, 2006). Since the study focuses on three a priori defined regions, including the amygdala, hippocampus and ACC, we extracted bilateral volumes for these specific regions. Total ACC volume for each hemisphere was calculated by adding the volume areas of the caudal and rostral sub-regions to avoid multiple testing issues. Intracranial volumes (ICV) were also generated by FreeSurfer and chosen as a covariate in the analyses to account for head size variability among participants. All T1 images were visually inspected by two independent researchers and images with clear motion artefacts (*N* = 3; patients) or incidental findings (*N* = 2; HCs (healthy controls)) were not included in the analysis. The exclusion of images with motion artefacts was also supported by the assessment of outliers using a standardised protocol provided by the ENIGMA (Enhancing Neuro Imaging Genetics through Meta-Analysis) consortium (http://enigma.ini.usc.edu/) as the images with significant motion artefacts returned a high number of errors in reconstruction (varying between 20 and 30).

### Statistical analysis

Statistical analyses were conducted with Statistical Package for Social Sciences (SPSS) Version 25 (SPSS IBM Corp., 2017) and the mediation analyses were performed with PROCESS macro Version 3.3 (Hayes, 2013). Log transformations were applied to reduce skewness of the childhood trauma variables that were not normally distributed. Sexual abuse and physical abuse were converted into dichotomous variables (0 = absent, 1 = present) since the majority of the sample (i.e. 86% and 80%, respectively) did not report these experiences. Where specified, years of education was controlled in the analyses due to significant group differences on this variable and its possible impact on brain structure and cognition (Cox *et al.*, 2016; Guerra-Carrillo *et al.*, 2017).

#### Between-group differences on childhood trauma, ROI volumes and emotion recognition.

To compare both groups on childhood trauma and emotion recognition measures, we first conducted independent sample *t*-tests and chi-square (χ^2^) tests, where appropriate. ANCOVAs (Analyses of covariance) were used to assess between-group differences on the specified brain regions with age, years of education and ICV as covariates in these analyses.

#### Associations between childhood trauma, ROI volumes and emotion recognition in all participants.

In the next step, partial correlations were conducted between childhood trauma (i.e. physical neglect and total childhood trauma), emotion recognition task and ROI volumes for all participants in order to assess the relationships between these measures and select the variables for the mediation analyses. Additional correlations were subsequently conducted for both groups separately (healthy participants and patients). Age, years of education and ICV were controlled for.

#### Moderated mediation analyses.

To explore whether regional brain volumes in the amygdala, hippocampus and ACC mediate the association between childhood trauma and performance on the ERT task, we conducted moderated mediation analyses using Model 59 from the PROCESS macro (Hayes, 2013), which allowed the inclusion of a mediator and a moderator in the same model. This particular model was used as it considers moderation effects on all paths (i.e. direct and indirect), compared to other more common models. We examined both direct and indirect (mediating) effects between childhood trauma and the ERT task, with the ROI volumes as mediators in these associations and a group type as a moderator, controlling for age, years of education and ICV. The reliability of the associated indirect effect was estimated using 5000 bootstrapping iterations to obtain bias-corrected and accelerated bootstrap 95% confidence intervals (CIs). When a 95% bootstrapped CI does not include zero, it indicates the parameter is statistically significant.

## Results

### Demographic and clinical data

Sociodemographic and clinical characteristics of the study participants are presented in Table 1. Patients did not significantly differ from healthy participants on age (*P* = 0.063) and gender (*P* = 0.838). As expected, both groups showed significant differences on years of education (*P* < 0.001) and estimated IQ (*P* < 0.001).

### Between-group differences on childhood trauma, emotion recognition and ROI volumes

Patients with SZ had significantly higher scores on physical neglect (*P* = 0.018), but not on emotional abuse (*P* = 0.187), physical abuse (*P* = 0.463), sexual abuse (*P* = 0.069) or emotional neglect (*P* = 0.560) compared to healthy participants (Table 1). Both groups also significantly differed on the total childhood trauma score (*P* = 0.049). As expected, patients showed significantly poorer performance than healthy participants on the ERT task (*P* < 0.001; Table 1).

When comparing patients and healthy participants on ROI volumes, we found that only the hippocampal volumes were significantly smaller in patients compared to healthy participants, including the total (*P* = 0.001) as well as the left and right hippocampal (*P* = 0.003; *P* = 0.001, respectively; Table 2) volumes. No significant differences on the amygdala and ACC volumes between patients and healthy participants were observed.

**Table 2.** Comparisons on ROI volumes between patients with SZ and healthy participants

	Patients (*N* = 46)	Healthy participants (*N* = 112)	Difference^a^
Variable	Mean (s.d.)	Mean (s.d.)	F/t
Amygdala (mm^3^)			
Left amygdala	1692.79 (226.04)	1736.84 (233.96)	0.454
Right amygdala	1833.77 (298.52)	1882.06 (251.54)	0.488
Total amygdala	3526.57 (488.48)	3618.89 (456.88)	0.567
Hippocampus (mm^3^)			
Left hippocampus	3978.57 (411.44)	4246.29 (394.74)	9.212**
Right hippocampus	3993.69 (420.39)	4284.17 (412.89)	11.683**
Total hippocampus	7972.29 (805.86)	8530.46 (780.42)	11.474**
ACC (mm^3^)			
Left ACC	4303.81 (818.70)	4465.72 (767.28)	0.076
Right ACC	3792.29 (669.81)	4019.02 (731.18)	1.079
Total ACC	8096.11 (1280.67)	8488.47 (1262.14)	0.202
*Intracranial volume (mm^3^)	1 502 170 (177 398)	1 536 520 (177 965)	1.109

ACC, anterior cingulate cortex.

^**^  *P* < 0.01.

^a^ Group differences on ROI volumes were analysed using ANCOVA and controlled for age, years of education and ICV.

### Associations between childhood trauma, emotion recognition and ROI volumes

#### All participants.

To assess the relationship between childhood trauma, emotion recognition and ROI volumes and to select the variables for the mediation analyses, partial correlations of these variables are reported for all participants (Table 3).

**Table 3.** Correlational analyses in all participants

Variable	ERT	PN	Total CTQ	Left amygdala	Right amygdala	Total amygdala	Left hip-pocampus	Right hip-pocampus	Total hip-pocampus	Left ACC	Right ACC	Total ACC
ERT	1.000	−0.321***	−0.134	0.046	0.084	0.073	0.168*	0.162*	0.172*	0.206*	0.224**	0.262**
PN	−0.321***	1.000	0.683***	−0.036	−0.084	−0.068	−0.074	−0.001	−0.037	−0.143	−0.115	−0.158^a^
Total CTQ	−0.134	0.683***	1.000	−0.066	−0.047	−0.061	−0.033	−0.005	−0.02	−0.138	−0.125	−0.161*
Left amygdala	0.046	−0.055	−0.036	1.000	0.664***	0.893***	0.574***	0.560***	0.592***	0.168*	0.074	0.150
Right amygdala	0.084	−0.141	−0.084	0.664***	1.000	0.929***	0.510***	0.444***	0.497***	0.189*	0.018	0.130
Total amygdala	0.073	−0.110	−0.068	0.893***	0.929***	1.000	0.590***	0.543***	0.592***	0.197*	0.047	0.152
Left hippocampus	0.168*	−0.074	−0.033	0.574***	0.510***	0.590***	1.000	0.831***	0.954***	0.069	0.080	0.091
Right hippocampus	0.162*	−0.001	−0.005	0.560***	0.444***	0.543***	0.831***	1.000	0.959***	0.107	0.062	0.104
Total hippocampus	0.172*	−0.037	−0.021	0.592***	0.497***	0.592***	0.954***	0.959***	1.000	0.093	0.074	0.102
Left ACC	0.206*	−0.143	−0.138	0.168*	0.189*	0.197*	0.069	0.107	0.093	1.000	0.339***	0.831***
Right ACC	0.224**	−0.115	−0.125	0.074	0.018	0.047	0.080	0.062	0.074	0.339***	1.000	0.805***
Total ACC	0.262**	−0.158^a^	−0.161*	0.150	0.130	0.152	0.091	0.104	0.102	0.831***	0.805***	1.000

ERT, Emotion Recognition Task; PN, physical neglect; CTQ, Childhood Trauma Questionnaire; ACC, anterior cingulate cortex.

*P < 0.05; **P < 0.01; ***P < 0.001.

^a^ Trend towards significance, *P* = 0.052.

**Fig. 1.**
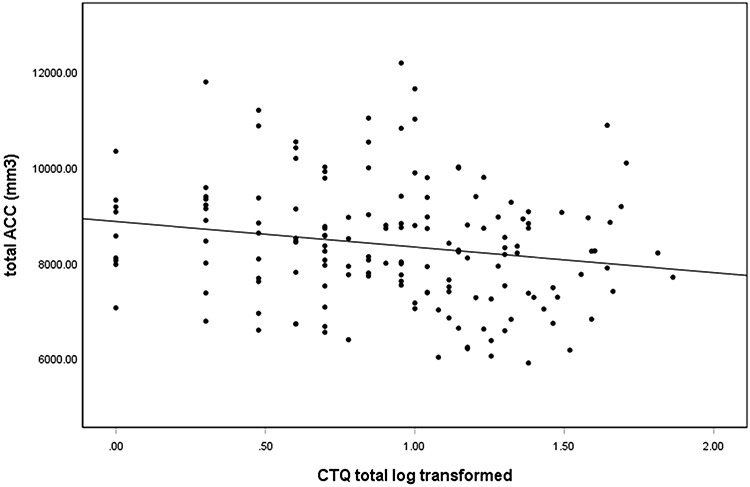
Association between the total ACC volume and CTQ total score (log transformed) in all participants. The scatter plot is unadjusted for covariates for illustration purposes (r = −.184, p = .022).

**Fig. 2.**
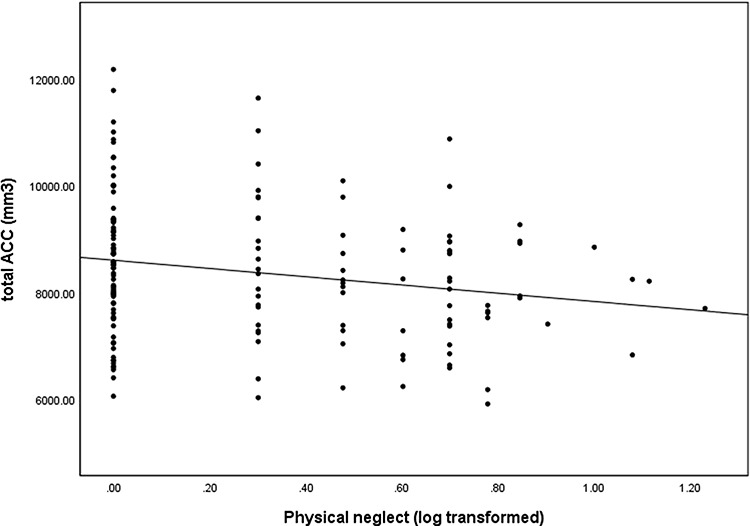
Association between the total ACC volume and physical neglect (log transformed) in all participants. The scatter plot is unadjusted for covariates for illustration purposes (r = −.204, p = .011).

Regarding the associations between ‘childhood trauma and ROI volumes’, CTQ total score was significantly negatively correlated with the total ACC volume (*r* = −0.161, *P* = 0.048) (Figure 1). There was also a trend towards significance for the association between physical neglect and the total ACC volume (*r* = −0.158, *P* = 0.052) (Figure 2). Total CTQ scores were also negatively correlated with hippocampal and amygdala volumes, but these correlations were non-significant.

In terms of the associations between ‘performance on the ERT task and the ROI volumes’, the ERT score was significantly positively associated with the total (*r* = 0.262, *P* = 0.001), left (*r* = 0.206, *P* = 0.011) and right (*r* = 0.224, *P* = 0.005) ACC volumes as well as the total (*r* = 0.172, *P* = 0.032), left (*r* = 0.168, *P* = 0.037) and right (*r* = 0.162, *P* = 0.044) hippocampal volumes. No significant correlations were reported between the amygdala and ERT task. The ERT score was also significantly negatively associated with physical neglect (*r* = −0.321, *P* < 0.001), but not total childhood trauma score (*r* = −0.134, *P* = 0.098).

#### Healthy participants.

In subsequent correlational analyses conducted in healthy participants only, physical neglect and CTQ total score were significantly negatively correlated with the left ACC volumes (*r* = −0.203, *P* = 0.037; *r* = −0.206, *P* = 0.034, respectively). Total CTQ score was also significantly negatively associated with the total ACC volumes (*r* = −0.227, *P* = 0.019). No significant associations were reported with hippocampal and amygdala volumes.

In terms of the association between performance on the ERT task and the ROI volumes, the ERT score was significantly positively correlated with the total (*r* = 0.313, *P* = 0.001), left (*r* = 0.233, *P* = 0.016) and right ACC volumes (*r* = 0.281, *P* = 0.003). No significant correlations were reported with hippocampal and amygdala volumes. The ERT score was also significantly negatively associated with physical neglect (*r* = −0.215, *P* = 0.025), but not total childhood trauma score (*r* = −0.175, *P* = 0.069).

#### Patients with SZ.

When conducting correlational analyses in patients only, physical neglect and CTQ total score were not significantly associated with any of the ROI volumes. However, a significant positive association was found between the ERT task and left hippocampal volume (*r* = 0.325, *P* = 0.034). The ERT score was also significantly negatively associated with physical neglect (*r *= −0.317, *P* = 0.038), but not total childhood trauma score (*r* = 0.131, *P* = 0.403).

Additionally, we examined the possible impact of antipsychotic medication on brain structure and behavioural performance in patients with SZ, however, we found no significant correlations, indicating that findings were independent of medication in our sample.

### Moderated mediation analyses

In order to test our main hypothesis regarding the direct and indirect effects of physical neglect and the CTQ total score on emotion recognition as mediated by volumetric changes, we tested two moderated mediation models in which group (healthy participants and patients separately) represented a moderator variable, and age, years of education and ICV were included as covariates. Based on the correlational analyses, only the ACC regions were entered as mediators in both models as they were associated with childhood trauma as well as emotion recognition measures. Physical neglect and the CTQ total score served as independent predictor variables and total ERT score was selected as the outcome (dependent) variable (Table 4). Additional analyses with the amygdala and hippocampal volumes as mediators were subsequently conducted to ascertain the possible mediating effects of these regions. A significant effect of diagnosis as a moderating variable was observed (*F* = 5.6; df1,148; *P* = 0.019); therefore, the mediating effects of brain volume are reported separately for healthy controls and patients.

**Table 4.** Moderated mediation analyses

	Direct effect of IV on DV				Indirect effect	
Independent variable (IV) × dependent variable (DV)	Group type	*b* (SE)	95% CI [LLCI : ULCI]	Mediating variable (moderated by the group type)	*b* (SE)	95% CI [LLCI : ULCI]
				Total ACC		
CTQ total score × ERT overall score	Healthy controls	−0.0207 (0.0346)	[−0.0890: 0.0477]	Healthy controls	−0.0206 (0.0101)	[−0.0437: −0.0045]^a^
	Patients	0.0082 (0.0391)	[−0.0691: 0.0855]	Patients	0.0112 (0.0153)	[−0.0087: 0.0499]
				Left ACC		
				Healthy controls	−0.0145 (0.0087)	[−0.0353: −0.0012]^a^
				Patients	0.0118 (0.0155)	[−0.0076: 0.0532]
				Right ACC		
				Healthy controls	−0.0119 (0.0092)	[−0.0356: −0.0001]^a^^,^^b^
				Patients	0.0037 (0.0095)	[−0.0099: 0.0290]
				Total ACC		
Physical neglect × ERT overall score	Healthy controls	−0.1803 (0.1633)	[−0.5030: 0.1425]	Healthy controls	−0.0692 (0.0431)	[−0.1779: −0.0083]^a^
	Patients	−0.5597 (0.1856)	[−0.9265: −0.1928]**	Patients	−0.0307 (0.0467)	[−0.1221: 0.0689]
				Left ACC		
				Healthy controls	−0.0643 (0.0425)	[−0.1699: −0.0034]^a^
				Patients	−0.0235 (0.523)	[−0.1278: 0.0924]
				Right ACC		
				Healthy controls	−0.0219 (0.0334)	[−0.1028: 0.0294]
				Patients	−0.0134 (0.0407)	[−0.0873: 0853]

SE = standard error; *b* = unstandardized coefficient; CI = confidence interval; LLCI = lower level confidence interval; ULCI = upper level confidence interval.

**P < 0.01.

^a^ Significant indirect effect as a 95% bootstrapped CI does not include zero.

^b^ After controlling for gender in addition to age, years of education and ICV, this finding did not remain significant.

#### Healthy participants.

**Fig. 3.**
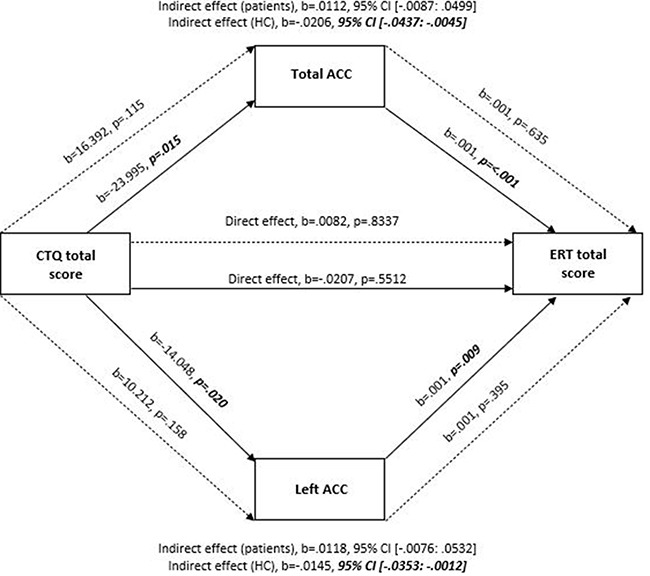
A simplified moderation mediation model on the mediating effects of the total and left ACC in the relationship between the CTQ and ERT total scores, controlling for age, years of education and ICV. Note: Solid lines represent healthy controls (HC) and dashed lines represent patients. All presented effects are unstandardized; b = unstandardized coefficient; CTQ: Childhood Trauma Questionnaire; ACC: anterior cingulate cortex; ERT: Emotion Recognition Task.

**Fig. 4.**
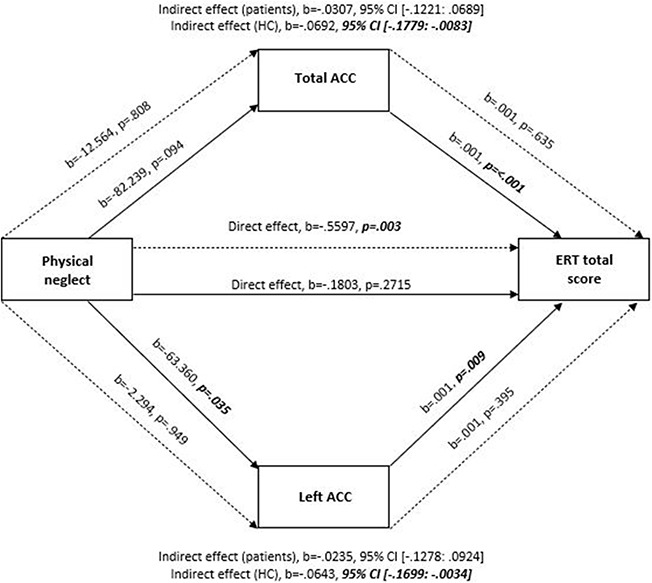
A simplified moderation mediation model on the mediating effects of the total and left ACC in the relationship between physical neglect and the ERT total score, controlling for age, years of education and ICV. Note: Solid lines represent healthy controls (HC) and dashed lines represent patients. All presented effects are unstandardized; b = unstandardized coefficient; ACC: anterior cingulate cortex; ERT: Emotion Recognition Task.

In healthy participants, significant indirect effects were observed in the association between the CTQ total score and the ERT task scores by total, left and right ACC volumes (Total ACC: *b* = −0.0206, SE = 0.0101, 95% CI [−0.0437: −0.0045]; Left ACC: b = −0.0145, SE = 0.0087, 95% CI [−0.0353: −0.0012] and Right ACC: *b* = −0.0119, SE = 0.0092, 95% CI [−0.0356: −0.0001], respectively; Table 4 and Figure 3). We also found evidence of full mediation of the association between physical neglect and ERT total scores by both total and left ACC volumes (Total ACC: *b* = −0.0692, SE = 0.0431, 95% CI [−0.1779: −0.0083] and Left ACC: *b* = −0.0643, SE = 0.0425, 95% CI [−0.1699: −0.0034]; Table 4 and Figure 4). In additional analyses with the amygdala and hippocampal volumes as mediators, no significant indirect effects were observed in healthy participants.

Following these mediating effects, the direct pathway of CTQ total score (*b* = −0.0207, SE = 0.0346, *P* = 0.5512, 95% CI [−0.0890: 0.0477]) and physical neglect score (*b* = −0.1803, SE = 0.1633, *P* = 0.2715, 95% CI [−0.5030: 0.1425]) on emotion recognition were not significant (indicating full mediation). After inclusion of gender as another covariate in addition to age, years of education and ICV, all results remained significant except for the mediating effects of the right ACC volume in the association between the CTQ total score and the ERT task.

#### Patients with SZ.

In patients, a significant direct association was found between physical neglect and the ERT task (*b* = −0.5597, SE = 0.1856, *P* = 0.003, 95% CI [−0.9265: −0.1928]), but not between the CTQ and ERT total scores (*b* = 0.0082, SE = 0.0391, *P* = 0.8337, 95% CI [−0.0691: 0.0855]) (Table 4, Figures 3 and 4). No indirect (mediating) effects were observed in this group for the ACC volumes. In additional analyses with the amygdala and hippocampal volumes as mediators, no significant indirect effects were observed in patients. After the inclusion of gender as another covariate in addition to age, years of education and ICV, direct effects of physical neglect on the ERT task remained significant.

## Discussion

In this study, we tested the hypothesis that the association between higher levels of childhood trauma and poorer emotion recognition would be mediated via reduced volumes in one or more of three stress-sensitive brain regions—the hippocampus, amygdala and/or ACC. We observed that the association between increased childhood trauma exposure (measured in terms of both physical neglect and total childhood trauma scores) and lower emotion recognition scores is mediated, at least partly, via reduced total and left ACC volumes in healthy participants. This was observed in the absence of similar associations with either the hippocampus or amygdala. To the best of our knowledge, this is the first study to evaluate a specific link between childhood trauma, brain structure and emotion recognition.

### The role of the ACC in the association between childhood trauma and emotion recognition

When examining the association between childhood trauma and the ROI volumes in the whole sample, we observed a significant negative association between physical neglect and the overall CTQ score, with the total ACC volumes. These results are consistent with previous studies reporting smaller ACC volumes in clinical and non-clinical populations following early life trauma (Dannlowski *et al.*, 2012; Meng *et al*., 2014; Cancel *et al.*, 2019). We also observed a significant positive association between emotion recognition and the total, left and right volumes of the ACC. Consistent with these findings, several studies have suggested that better ability to recognise emotions is associated with larger ACC volumes in both clinical and non-clinical populations (Fujiwara *et al.*, 2007; Szymkowicz *et al.*, 2016).

In terms of our main hypothesis that childhood trauma related effects on emotion recognition would be mediated by volumetric changes, reduced ACC volumes were observed to fully mediate the association between childhood trauma and emotion recognition in healthy participants. The role of the ACC region as a potential mediator in the relationship between childhood trauma and emotion recognition is novel and highlights the importance of this particular brain area in emotion processing.

There are a range of possible explanations for why variation of volume in the ACC may mediate the effects of childhood trauma on emotion recognition, including (i) the idea of a trauma-sensitive critical period for brain development, (ii) the role of the ACC in emotional awareness and (iii) the role of the ACC in detecting social exclusion. In terms of the notion that the ACC region may be more sensitive to adversity during development, some researchers (Do *et al.*, 2009; Pizzagalli, 2011; Teicher *et al.*, 2016) argue that there may be a ‘critical period’ for the normal development of anterior cortical areas, during which the impact of various stressors could be particularly detrimental. The PFC is a region with delayed ontogeny (Weinberger *et al.*, 1986), and consequently may be affected more by environmental stressors than other brain areas, as shown in studies of animal models (Radley *et al.*, 2006; Pascual and Zamora-Leon, 2007). Another possible explanation for a potential mediating role of the ACC is that this region is involved in emotional awareness and self-referential thinking essential for conscious self-monitoring, which in turn is important for adapting cognitively and socially to various experiences and being aware of the environment (Lou *et al.*, 2017). Trauma-related alterations to ACC may impair these processes, affecting the ability to accurately identify emotional expressions. Further, ACC has also been shown to be a key region involved in the detection of social exclusion. Since humans are sensitive about being included by others, negative experiences associated with rejection (e.g. physical neglect) may affect the ability to effectively adapt to social environments by hindering monitoring or detection systems that are highly sensitive to social exclusion (Gauthier *et al.*, 1996; Kawamoto *et al.*, 2012; Garcia *et al.*, 2016). The ACC region is in fact often implicated in physical and social pain research as disruption in ACC activity has been consistently demonstrated while experiencing physical as well as ‘social pain’, such as rejection, exclusion, humiliation or grief, based on previous studies and meta-analyses (Eisenberger *et al.*, 2003; Eisenberger, 2015; Lieberman and Eisenberger, 2015; Rotge *et al.*, 2015).

In regard to our clinical sample, we did not find the same mediating role of the ACC in patients. This was despite the fact that our patient sample showed higher rates of childhood trauma experiences (specifically physical neglect and total CTQ scores). Given the previously observed association between childhood trauma, structural brain alterations and poor social cognitive function in patients with SZ (Trémeau, 2006; Rokita *et al.*, 2018, 2020; Cancel *et al.*, 2019; Popovic *et al.*, 2019), the lack of significant mediating effects in the patient group is surprising. One potential explanation for this difference between healthy participants and patients relates to the difference in sample size between these groups. While the patient group was relatively large for a sample of this size, it is difficult to calculate exactly what the power of a given sample is in mediation analysis. This, together with the known heterogeneity in patients with SZ, in terms of illness severity (Wolfers *et al.*, 2018), brain structure (Zhang *et al.*, 2014) and cognitive performance (Carruthers *et al.*, 2019), may have prevented us from being able to detect indirect (mediating) associations, despite the high levels of childhood adversity reported.

### The role of the amygdala and hippocampus in the association between childhood trauma and emotion recognition

In terms of an association between childhood trauma and the other two ROIs in the whole sample, we did not observe significant associations between childhood trauma and amygdala volumes, contrary to findings from Aas *et al.* (2012) and Hoy *et al.* (2012). However, several meta-analyses showed inconsistent findings for the amygdala (McCrory *et al.*, 2011; O’Doherty *et al.*, 2015). In fact, volumetric changes of the amygdala are complex and likely to be moderated by the type and exposure timing of childhood trauma (Berens *et al.*, 2017). We also did not observe significant associations between amygdala volume and the ability to recognise emotions. The importance of this brain region in emotion processing has been demonstrated primarily in functional MRI studies rather than structural imaging studies (Taylor *et al.*, 2012; Spohrs *et al.*, 2018; Zinchenko *et al.*, 2018). Where changes in amygdala volume have been correlated with emotion recognition, these associations have been specific to negative facial expressions (e.g. fearful) (Zhao *et al.*, 2013; Warnell *et al.*, 2018), whereas our study examined the ability to recognise emotions more generally.

Similar to Aas *et al.* (2012), we did not find a significant association between childhood trauma and hippocampal volumes. However, we did observe a significant positive association between the ERT total score and the total, left and right volumes of the hippocampus, which is consistent with previous studies (Fujiwara *et al.*, 2007; Szymkowicz *et al.*, 2016). In terms of our mediation hypothesis, the hippocampus was not observed to mediate the effects of childhood trauma on emotion recognition in either group. The lack of mediating effects of the hippocampus may reflect the influence of other factors that were not examined in this study. For instance, school/workplace bullying and other stressful events in adulthood have also been associated with variations in hippocampal volume (Zannas *et al.*, 2013; Nolfe *et al.*, 2018).

### Between-group differences on ROI volumes

When examining between-group differences on ROI volumes, we observed significant reductions of the total, left and right hippocampal volumes in patients compared to healthy participants. This is one of the most consistently reported findings in SZ. Numerous neuroimaging studies and meta-analyses have reported reduced hippocampal volumes in chronic patients as well as those with first-episode psychosis and ultra-high risk individuals (Wright *et al.*, 2000; Sumich *et al.*, 2002; Arnold *et al.*, 2015; Dean *et al.*, 2015; Ho *et al.*, 2017; Lieberman *et al.*, 2018). In terms of between-group differences on the amygdala and ACC volumes, no significant differences were reported. Considering the effect sizes reported for these differences by the ENIGMA Schizophrenia Working Group (van Erp *et al.*, 2016), the non-significance of these differences is likely to reflect the limited power in the current sample.

### Limitations

Although the findings of our study provide novel evidence for the detrimental effects of childhood trauma on emotion processing and structural brain measures of volume associated with emotion recognition deficits, some limitations should be mentioned. Firstly, the assessment of childhood trauma experiences was based on a retrospective self-report. Although the CTQ questionnaire is reported to provide reliable and valid screening of childhood maltreatment experiences (Bernstein *et al.*, 1997), such measures are generally criticised for liability to recall bias. Based on our observations during testing, it appears that a number of participants, specifically patients, may have under-reported their early life traumatic experiences as feedback from some individuals seems to suggest that they felt uncomfortable disclosing childhood experiences. Such under-reporting of traumatic experiences may have impacted our ability to accurately measure the mediating effects of brain structure in relation to childhood trauma and emotion recognition. Several studies have suggested that patients with SZ are more likely to under-report instances of childhood maltreatment than healthy individuals for a variety of reasons, such as distrust towards researchers or other professionals, not feeling comfortable disclosing information on this sensitive topic or to avoid pain and embarrassment when verbalising and thinking about these experiences—the former is likely to be related to SZ symptoms (e.g. paranoia) (Fisher *et al.*, 2009; McKinney *et al.*, 2009). In future studies, the accuracy of childhood maltreatment measures might be aided by the use of additional sources, such as clinical documentation or family interviews, where possible. Secondly, because of a multiple testing burden, we did not include other brain regions involved in emotion recognition impairments in our analysis, for instance the insula or the fusiform face area, which are also relevant to understanding the relationship between childhood trauma and emotion recognition studied here. Thirdly, we did not assess other stressful life events, including traumatic experiences after the age of 18, which may also contribute to structural changes in several brain regions, including the hippocampus, amygdala and ACC (Papagni *et al.*, 2011). Further, it is possible that larger samples of patients with SZ would yield more prominent findings, similar to those observed in the healthy group. Additionally, our study examined the effects of childhood trauma on brain structure and emotion recognition in a mixed patient sample consisting of individuals with SZ and those with a SZA. Further studies should examine this relationship in both groups separately to determine the possible differential outcomes. Lastly, we were unable to investigate the possible impact of childhood trauma duration and onset, which could contribute to the results as there may be sensitive periods when specific brain areas are most sensitive to stressful events (Dannlowski *et al.*, 2012; Gee and Casey, 2015).

## Conclusion

In conclusion, our findings provide further evidence of an association between childhood trauma, specifically physical neglect and total childhood trauma, emotion recognition and variation in brain volume in stress- and emotion-associated brain regions in both healthy participants and patients with SZ. We also provide novel evidence that variation in ACC volumes may mediate the association observed between childhood trauma and the ability to recognise emotions, suggesting that one potential mechanism by which childhood trauma may exert a deleterious effect on social cognitive function is via specific brain areas. If replicated, these findings have important implications for clinical and translational research as they underline the importance of early interventions (e.g. parenting or general community-wide programmes) aimed at preventing or reducing childhood maltreatment as well as promoting healthy child development and positive parenting. Since childhood trauma experiences have a particularly high prevalence in people with psychiatric disorders and thus, are considered a risk factor for mental health problems, development and implementation of these interventions are of crucial importance.

Future studies should investigate the possible role of genetic variation combined with childhood trauma in contributing to both changes in brain structure and neurocognitive function in both clinical and non-clinical populations. So doing is likely to be highly informative in the further development of early intervention strategies aiming to mitigate the detrimental effects of trauma.

## Supplementary Material

nsaa160_Supp
