# Supplementary material for: Childhood trauma, brain structure and emotion recognition in patients with schizophrenia and healthy participants
Source: Soc Cogn Affect Neurosci. 2020 Nov 27;15(12):1325–39. doi: 10.1093/scan/nsaa160 (PMC7759212; doi:10.1093/scan/nsaa160)
Supplement: nsaa160_Supp [file nsaa160_supp.zip › Supplementary.docx]

**Supplementary Table 1.** Studies linking childhood trauma to structural brain abnormalities (specifically in the amygdala, hippocampus and ACC) in psychiatric and healthy populations.

| Authors | Group | Childhood trauma evaluation | Main results |
| --- | --- | --- | --- |
| Herzog *et al.* (2020) | HC, N=68 (females only) | MACE | Significant association was found between global ACE severity and bilateral amygdala volume. Severe experiences of neglect were associated with smaller bilateral amygdala volume, and at trend level with smaller bilateral hippocampal volume across traumatized individuals. |
| Teicher *et al.* (2018) | HC, N=336 | MACE | Childhood abuse and neglect significantly predicted reduced hippocampal volumes. |
| Aas *et al.* (2014) | SZ, N=31;  SZA, N=4;  Other psychoses, N=15;  BD, N=55;  MDD, N=2 | CTQ | *Met* carriers of the BDNF *val66met* had reduced volume of the hippocampal subfields, specifically for CA2/3 and the CA4 DG. The strongest associations were found for patients with a history of sexual abuse and physical abuse. |
| Kumari *et al.* (2014) | ASPD, N=14;  SZ, N=28;  HC, N=15 | Psychosocial deprivation interview | ACC volumes correlated significantly negatively with total psychosocial deprivation ratings as well as with ratings of sexual and physical abuse. |
| Opel *et al.* (2014) | MDD, N=85;  HC, N=85 | CTQ | Childhood maltreatment was significantly associated with hippocampal volume loss in both patients and healthy controls. |
| Baker *et al.* (2013) | HC, N=173 | ELSQ | Later childhood ELS (8 – 17 years) was associated with volumetric reductions in the ACC and insula volumes, while ELS experienced in early childhood (between the ages of 1 month and 7 years) was not associated with lower brain volumes in these regions. |
| Heim *et al.* (2013) | HC, N=51(females only) | CTQ | Childhood emotional abuse was associated with cortical thinning in the left and right precuneus, left ACC, right parahippocampal gyrus, and left somatosensory cortex. |
| Aas *et al.* (2012) | FEP, N=86;  HC, N=63 | CECAQ | A history of childhood trauma was associated with smaller amygdala volume. |
| Carballedo *et al.* (2012) | HC with family history of depression, N=20;  HC with no family history of depression, N=20 | CTQ | FHP subjects, who had a previous history of childhood emotional abuse, showed smaller volumes in the left MPFC, left DLPFC and ACC compared to FHP without history of childhood abuse |
| Dannlowski *et al.* (2012) | HC, N=148 | CTQ | Reduced gray matter volumes in the hippocampus, insula, orbitofrontal cortex, ACC, and caudate were significantly associated with high CTQ scores. |
| Hoy *et al.* (2012) | FEP, N=21 | TEC | The experience of childhood trauma was significantly associated with hippocampal and amygdala volumes. |
| Malykhin *et al.* (2012) | MDD, N=39 | CTQ | Reduced ACC volumes were related to severe abuse. |
| Teicher *et al.* (2012) | HC, N=193 | CTQ | The experience of childhood maltreatment was significantly associated with reduced hippocampal volume, specifically in the left CA2-CA3 and CA4-DG subfields. |
| Driessen *et al.* (2000) | BPD, N=21;  HC, N=21 | CTQ | Hippocampal volumes were negatively significantly associated with early traumatic experiences across the sample. |
| Frodl *et al.* (2010) | MDD, N=43;  HC, N=44 | CTQ | A history of childhood emotional neglect was significantly associated with reduced left hippocampal volume in patients, compared to those with no such experiences. Physical neglect was significantly associated with reduced gray matter volume in healthy subjects. |
| Thomaes *et al.* (2010) | PTSD, N=31;  HC, N=28 | STI | Patients with child abuse-related complex PTSD showed reductions in gray in right hippocampus and right dorsal ACC compared to controls. |
| Tomoda *et al.* (2009) | HC, N=1455 | LEQ | Subjects with harsh corporal punishment during childhood were reported to have significant reductions in grey matter volume in the right medial frontal gyrus, left medial frontal gyrus and in the right anterior cingulate gyrus. |
| Treadway *et al.* (2009) | MDD, N=18;  HC, N=18 | CTQ | A history of childhood trauma in patients with MDD was significantly negatively associated with the ACC volumes. |
| Weniger *et al.* (2009) | PTSD, N=10;  DA/DID, N=13;  HC, N=25 | TAQ | Trauma‐exposed subjects with PTSD displayed significantly reduced amygdala and hippocampal size, compared to patients with DA/DID and controls. |
| Andersen *et al.* (2008) | Abused females, N=26;  HC, N=17 | TAQ | Hippocampal volume was reduced in association with childhood sexual abuse at 3–5 years and 11–13 years. |
| Cohen *et al.* (2006) | HC, N=265 | ELSQ | Participants with greater than two ACEs had smaller ACC and caudate nuclei than those without ACEs. |
| Vermetten *et al.* (2006) | DID, N=15;  HC, N=23 | ETI | Significantly smaller hippocampal and amygdalar volumes were observed in patients with DID with a self-report of childhood trauma. |
| Brambilla *et al.* (2004) | BPD, N=10;  HC, N=20 | Abuse history questionnaire | Compared with healthy controls, BPD subjects with a reported history of childhood abuse had significantly smaller right and left hippocampal volumes. |
| Bremner *et al.* (2003) | PTSD, N=10 (females only);  HC, N=23 (females only) | ETI | Women with PTSD with a history of childhood abuse had significantly smaller hippocampus compared to women with abuse without PTSD and controls. |
| Schmahl *et al.* (2003) | BPD, N=10 (females only);  HC, N=23 (females only) | ETI | Patients with a history of childhood trauma had smaller amygdala and hippocampal volumes, compared to controls. |
| Vythilingam *et al.* (2002) | MDD, N=32;  HC, N=14 | ETI | Patients with MDD with a history of childhood abuse had significantly smaller hippocampal volumes than the non-abused patients and controls. |
| Bremner *et al.* (1997) | PTSD, N=17;  HC, N=17 | ETI | PTSD patients with a history of childhood abuse had a smaller left hippocampal volume relative to the matched controls. |
| Stein *et al.* (1997) | PTSD, N=21 (females only);  HC, N=21 (females only) | Telephone interview | Women with PTSD who reported sexual abuse in childhood had significantly reduced left-sided hippocampal volume compared to the non-abused controls. |

Abbreviations; ACC: anterior cingulate cortex; ACE: adverse childhood experiences; ASPD: Antisocial Personality Disorder; BD: Bipolar Disorder; BPD: Borderline Personality Disorder; CECAQ: Childhood Experience of Care and Abuse Questionnaire; CTQ: Childhood Trauma Questionnaire; DA: Dissociative Amnesia; DID: Dissociative Identity Disorder; ELS: early life stress; ELSQ: Early Life Stress Questionnaire; ETI: Early Trauma Inventory; FEP: First-episode Psychosis; HC: healthy controls; LEQ: Life Experiences Questionnaire; MACE: Maltreatment and Abuse Chronology of Exposure Interview; MDD: Major Depressive Disorder; PTSD: Posttraumatic Stress Disorder STI: Structured Trauma Interview; TAQ: Traumatic Antecedent Questionnaire; TEC: Traumatic Experiences Checklist

**References**

Aas, M., Haukvik, U. K., Djurovic, S., Tesli, M., Athanasiu, L., Bjella, T., Hansson, L., Cattaneo, A., Agartz, I., Andreassen, O. A. & Melle, I. (2014) 'Interplay between childhood trauma and BDNF val66met variants on blood BDNF mRNA levels and on hippocampus subfields volumes in schizophrenia spectrum and bipolar disorders', *J Psychiatr Res*, **59**, pp. 14-21.

Aas, M., Navari, S., Gibbs, A., Mondelli, V., Fisher, H. L., Morgan, C., Morgan, K., MacCabe, J., Reichenberg, A., Zanelli, J., Fearon, P., Jones, P. B., Murray, R. M., Pariante, C. M. & Dazzan, P. (2012) 'Is there a link between childhood trauma, cognition, and amygdala and hippocampus volume in first-episode psychosis?', *Schizophr Res*, **137**(1-3), pp. 73-79.

Andersen, S. L., Tomada, A., Vincow, E. S., Valente, E., Polcari, A. & Teicher, M. H. (2008) 'Preliminary evidence for sensitive periods in the effect of childhood sexual abuse on regional brain development', *J Neuropsychiatry Clin Neurosci*, **20**(3), pp. 292-301.

Baker, L. M., Williams, L. M., Korgaonkar, M. S., Cohen, R. A., Heaps, J. M. & Paul, R. H. (2013) 'Impact of early vs. late childhood early life stress on brain morphometrics', *Brain Imaging Behav*, **7**(2), pp. 196-203.

Brambilla, P., Soloff, P. H., Sala, M., Nicoletti, M. A., Keshavan, M. S. & Soares, J. C. (2004) 'Anatomical MRI study of borderline personality disorder patients', *Psychiatry Research: Neuroimaging*, **131**(2), pp. 125-133.

Bremner, J. D., Randall, P., Vermetten, E., Staib, L., Bronen, R. A., Mazure, C., Capelli, S., McCarthy, G., Innis, R. B. & Charney, D. S. (1997) 'Magnetic resonance imaging-based measurement of hippocampal volume in posttraumatic stress disorder related to childhood physical and sexual abuse--a preliminary report', *Biol Psychiatry*, **41**(1), pp. 23-32.

Bremner, J. D., Vythilingam, M., Vermetten, E., Southwick, S. M., McGlashan, T., Nazeer, A., Khan, S., Vaccarino, L. V., Soufer, R., Garg, P. K., Ng, C. K., Staib, L. H., Duncan, J. S. & Charney, D. S. (2003) 'MRI and PET study of deficits in hippocampal structure and function in women with childhood sexual abuse and posttraumatic stress disorder', *Am J Psychiatry*, **160**(5), pp. 924-932.

Carballedo, A., Lisiecka, D., Fagan, A., Saleh, K., Ferguson, Y., Connolly, G., Meaney, J. & Frodl, T. (2012) 'Early life adversity is associated with brain changes in subjects at family risk for depression', *World J Biol Psychiatry*, **13**(8), pp. 569-578.

Cohen, R. A., Grieve, S., Hoth, K. F., Paul, R. H., Sweet, L., Tate, D., Gunstad, J., Stroud, L., McCaffery, J., Hitsman, B., Niaura, R., Clark, C. R., MacFarlane, A., Bryant, R., Gordon, E. & Williams, L. M. (2006) 'Early Life Stress and Morphometry of the Adult Anterior Cingulate Cortex and Caudate Nuclei', *Biol Psychiatry*, **59**(10), pp. 975-982.

Dannlowski, U., Stuhrmann, A., Beutelmann, V., Zwanzger, P., Lenzen, T., Grotegerd, D., Domschke, K., Hohoff, C., Ohrmann, P., Bauer, J., Lindner, C., Postert, C., Konrad, C., Arolt, V., Heindel, W., Suslow, T. & Kugel, H. (2012) 'Limbic scars: long-term consequences of childhood maltreatment revealed by functional and structural magnetic resonance imaging', *Biol Psychiatry*, **71**(4), pp. 286-293.

Driessen, M., Herrmann, J., Stahl, K., Zwaan, M., Meier, S., Hill, A., Osterheider, M. & Petersen, D. (2000) 'Magnetic Resonance Imaging Volumes of the Hippocampus and the Amygdala in Women With Borderline Personality Disorder and Early Traumatization', *Arch Gen Psychiatry*, **57**(12), pp. 1115-1122.

Frodl, T., Reinhold, E., Koutsouleris, N., Reiser, M. & Meisenzahl, E. M. (2010) 'Interaction of childhood stress with hippocampus and prefrontal cortex volume reduction in major depression', *J Psychiatr Res*, **44**(13), pp. 799-807.

Heim, C. M., Mayberg, H. S., Mletzko, T., Nemeroff, C. B. & Pruessner, J. C. (2013) 'Decreased cortical representation of genital somatosensory field after childhood sexual abuse', *Am. J. Psychiatry American Journal of Psychiatry*, **170**(6), pp. 616-623.

Herzog, J. I., Thome, J., Demirakca, T., Koppe, G., Ende, G., Lis, S., Rausch, S., Priebe, K., Müller-Engelmann, M., Steil, R., Bohus, M. & Schmahl, C. (2020) 'Influence of Severity of Type and Timing of Retrospectively Reported Childhood Maltreatment on Female Amygdala and Hippocampal Volume', *Scientific Reports*, **10**(1), p. 1903.

Hoy, K., Barrett, S., Shannon, C., Campbell, C., Watson, D., Rushe, T., Shevlin, M., Bai, F., Cooper, S. & Mulholland, C. (2012) 'Childhood trauma and hippocampal and amygdalar volumes in first-episode psychosis', *Schizophr Bull*, **38**(6), pp. 1162-1169.

Kumari, V., Uddin, S., Premkumar, P., Young, S., Gudjonsson, G. H., Raghuvanshi, S., Barkataki, I., Sumich, A., Taylor, P. & Das, M. (2014) 'Lower anterior cingulate volume in seriously violent men with antisocial personality disorder or schizophrenia and a history of childhood abuse', *Aust N Z J Psychiatry*, **48**(2), pp. 153-161.

Malykhin, N. V., Carter, R., Hegadoren, K. M., Seres, P. & Coupland, N. J. (2012) 'Fronto-limbic volumetric changes in major depressive disorder', *J Affect Disord*, **136**(3), pp. 1104-1113.

Opel, N., Redlich, R., Zwanzger, P., Grotegerd, D., Arolt, V., Heindel, W., Konrad, C., Kugel, H. & Dannlowski, U. (2014) 'Hippocampal atrophy in major depression: a function of childhood maltreatment rather than diagnosis?', *Neuropsychopharmacology*, **39**(12), pp. 2723-2731.

Schmahl, C. G., Vermetten, E., Elzinga, B. M. & Douglas Bremner, J. (2003) 'Magnetic resonance imaging of hippocampal and amygdala volume in women with childhood abuse and borderline personality disorder', *Psychiatry Res*, **122**(3), pp. 193-198.

Stein, M. B., Koverola, C., Hanna, C., Torchia, M. G. & McClarty, B. (1997) 'Hippocampal volume in women victimized by childhood sexual abuse', *Psychol Med*, **27**(4), pp. 951-959.

Teicher, M. H., Anderson, C. M., Ohashi, K., Khan, A., McGreenery, C. E., Bolger, E. A., Rohan, M. L. & Vitaliano, G. D. (2018) 'Differential effects of childhood neglect and abuse during sensitive exposure periods on male and female hippocampus', *Neuroimage*, **169**, pp. 443-452.

Teicher, M. H., Anderson, C. M. & Polcari, A. (2012) 'Childhood maltreatment is associated with reduced volume in the hippocampal subfields CA3, dentate gyrus, and subiculum', *Proc Natl Acad Sci U S A*, **109**(9), pp. E563-572.

Thomaes, K., Dorrepaal, E., Draijer, N., de Ruiter, M. B., van Balkom, A. J., Smit, J. H. & Veltman, D. J. (2010) 'Reduced anterior cingulate and orbitofrontal volumes in child abuse-related complex PTSD', *J Clin Psychiatry*, **71**(12), pp. 1636-1644.

Tomoda, A., Suzuki, H., Rabi, K., Sheu, Y.-S., Polcari, A. & Teicher, M. H. (2009) 'Reduced prefrontal cortical gray matter volume in young adults exposed to harsh corporal punishment', *Neuroimage*, **47 Suppl 2**(Suppl 2), pp. T66-T71.

Treadway, M. T., Grant, M. M., Ding, Z., Hollon, S. D., Gore, J. C. & Shelton, R. C. (2009) 'Early Adverse Events, HPA Activity and Rostral Anterior Cingulate Volume in MDD', *PLoS One*, **4**(3), p. e4887.

Vermetten, E., Schmahl, C., Lindner, S., Loewenstein, R. J. & Bremner, J. D. (2006) 'Hippocampal and amygdalar volumes in dissociative identity disorder', *Am J Psychiatry*, **163**(4), pp. 630-636.

Vythilingam, M., Heim, C., Newport, J., Miller, A. H., Anderson, E., Bronen, R., Brummer, M., Staib, L., Vermetten, E., Charney, D. S., Nemeroff, C. B. & Bremner, J. D. (2002) 'Childhood trauma associated with smaller hippocampal volume in women with major depression', *Am J Psychiatry*, **159**(12), pp. 2072-2080.

Weniger, G., Lange, C., Sachsse, U. & Irle, E. (2009) 'Reduced amygdala and hippocampus size in trauma-exposed women with borderline personality disorder and without posttraumatic stress disorder', *J Psychiatry Neurosci*, **34**(5), pp. 383-388.
